# Obstectrical Teaching in Europe and America

**Published:** 1909-11

**Authors:** B. C. Hirst

**Affiliations:** Philadelphia, Pa.


### Obstectrical Teaching in Europe and America.

Philadelphia, Pa., October 19, 1909.

*Editor Buffalo Medical Journal:*

*Sir*—The president of the American Gynecological Society has appointed a committee to report at the next annual meeting in Washington, on the present status of obstetrical teaching in Europe and America, and to recommend improvements in the scope and character of the teaching of obstetrics in America.

The committee consists of the professors of obstetrics in Columbia University, University of Pennsylvania, Harvard, Jefferson Medical College, John Hopkins University, Cornell University and the University of Chicago.

Communications from anyone interested in the subject will be gladly received by the chairman of the committee, Dr. B. C. Hirst, 1821 Spruce Street, Philadelphia, Pa.

B. C. Hirst.

---

If the percentage of tuberculous children recently ascertained by an investigation in Stockholm, Sweden (1.61%) were applied to the schools of the United States, there would be 273,700 children between the ages of 8 and 15 who are positively affected with tuberculosis, according to a statement issued today by the National Association for the Study and Prevention of Tuberculosis. As contrasted with this figure, there are only eleven open air tuberculosis schools in the entire country.

Special schools for tuberculous children have now been established in Providence, Boston, New York, Rochester, Washington, Hartford, Conn., Chicago and Pittsburg. New York has three schools and Washington; D. C., two. The Board of Education of New York city is proposing to establish three more, and similar institutions are being planned in Detroit, Buffalo, Philadelphia, Cincinnati, and Newark, N. J.

At the lowest estimate, however, even with all the schools now in operation and those proposed, accommodations will not be provided for .4 of 1 per cent. of the children who need this special treatment. In a large number of cities, children with tuberculosis are excluded from the public schools, but in most instances, no special provision is made for them. The National Association declares that children who are afflicted with tuberculosis are a menace to the health of their schoolmates. Both on this account and because they are physically unable to keep up in their work, special schools are needed for this class of children. Every city should provide at least one well-equipped school or special class room of this sort for each 25,000 population.

